# Adjunctive Use of Point of Care Ultrasound to Diagnose Compartment Syndrome of the Thigh

**DOI:** 10.24908/pocus.v6i2.15185

**Published:** 2021-11-23

**Authors:** Neil Long, Justin S Ahn, Daniel J Kim

**Affiliations:** 1 Department of Emergency Medicine, Burnaby General Hospital Burnaby, British Columbia Canada; 2 Department of Emergency Medicine, University of British Columbia Vancouver , British Columbia Canada; 3 Department of Emergency Medicine, Royal Columbian Hospital New Westminster, British Columbia Canada; 4 Department of Emergency Medicine, Vancouver General Hospital Vancouver , British Columbia Canada

**Keywords:** compartment syndrome, thigh, point of care ultrasound

## Abstract

Compartment syndrome is a medical emergency and must be considered in patients who present with severe limb pain. Compartment syndrome is a clinical diagnosis, classically described as presenting with the 5 ‘P’s (pain, pulselessness, pallor, paraesthesia, and paralysis). Apart from pain, the other findings signify acute arterial obstruction and would be late findings. We present a case of a 31-year-old male in which point of care ultrasound (POCUS) expedited this diagnosis by demonstrating a large thigh hematoma in the anterior compartment. This prompted emergent orthopedic surgery consultation, and the diagnosis of compartment syndrome was confirmed both at the bedside and in the operating room. Compartment syndrome can be a challenging diagnosis, especially early in the course of illness. While POCUS should not be used in isolation in the assessment of possible compartment syndrome, it can be used as an adjunct in the workup, especially if it identifies an underlying cause.

## Introduction

Compartment syndrome is an emergency condition that while uncommon should be part of any emergency physician’s differential when presented with a patient in severe limb pain. If left untreated, irreversible muscle necrosis can occur resulting in loss of limb or life-threatening hyperkalemia resulting in ventricular dysrhythmias and potentially death. Compartment syndrome is a clinical diagnosis, classically characterized by the 5 ‘P’s (pain, pulselessness, pallor, paresthesia, and paralysis). However, in this case report we present a case where only a few of the classic findings were present, and the use of point of care ultrasound (POCUS) expedited diagnosis and management. 

## Case Report

A 31-year-old male presented to the emergency department (ED) with acute thigh pain and swelling. He had been struck as a pedestrian by a car at low speed 10 days prior, with the main impact to his right thigh. He was assessed in the ED at that time, diagnosed with minor soft tissue contusions, and discharged home. He had been having minimal discomfort, but that morning, he developed sudden onset, severe right thigh pain and swelling, and an inability to bear weight. His past medical history included von Willebrand disease and Noonan syndrome complicated by pulmonary stenosis. He was on no medications. On physical examination, he was afebrile with normal vital signs but appeared to be in moderate discomfort secondary to pain. The right thigh was swollen and firm, particularly over the anterior compartment. There was no redness or warmth. Severe pain was elicited with passive knee flexion and extension, but more so with knee flexion. The extremity remained neurovascularly intact.

**Figure 1:**
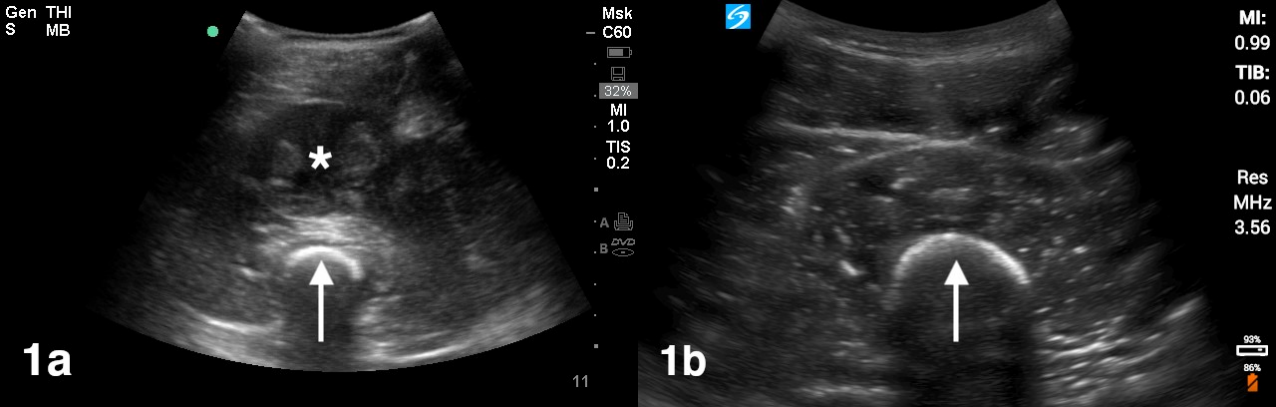
a) Ultrasound image of a large hematoma (asterisk) in the anterior thigh, appearing as a well circumscribed collection of mixed echogenicity. The femur (arrow) is the hyperechoic structure with posterior shadowing. b) Ultrasound image of a normal anterior thigh demonstrating normal sonographic muscle architecture with the femur (arrow) in the far field.

**Figure 2:**
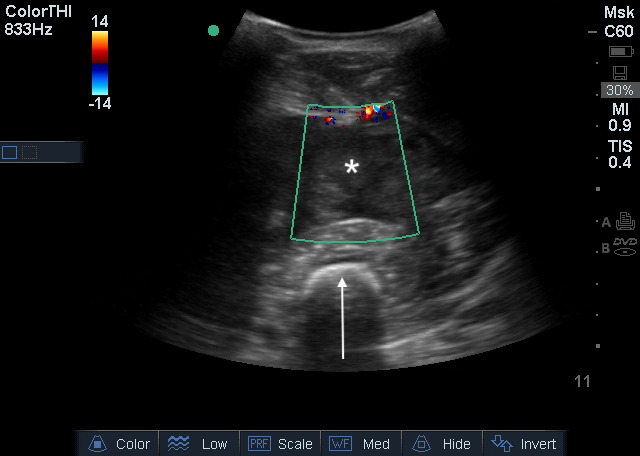
Ultrasound color Doppler image of a large hematoma (asterisk) in the anterior thigh, appearing as a well circumscribed collection of mixed echogenicity without vascularity. The femur (arrow) is the hyperechoic structure with posterior shadowing.

POCUS demonstrated a well circumscribed collection of mixed echogenicity in the anterior thigh consistent with a hematoma measuring 3.6 cm in the anteroposterior dimension, 8.2 cm in the transverse dimension, and 11.5 cm in the longitudinal dimension. The typical muscular anatomy of the thigh appeared distorted as a result (Figure 1a, online Video S1) compared to the normal contralateral thigh. (Figure 1b). On color Doppler, there was no increase in vascularity either within or surrounding the hematoma (Figure 2, online Video S2).

Orthopedic surgery was emergently consulted, and the orthopedic team performed compartment pressure measurements with a Stryker device. The pressure in the anterior compartment was >65 mm Hg. The patient was brought to the operating room and underwent urgent fasciotomy of the right anterior and posterior compartments. Once the fascia lata was opened, the vastus lateralis muscle bulged out through the fascial split, reflecting the abnormally high pressure in the anterior compartment. Although the musculature in the anterior compartment was bruised, it appeared healthy with normal color and contractility, speaking to the timeliness of the diagnosis and operative management. The patient required a delayed skin graft, and ultimately made a full recovery.

## Discussion

Compartment syndrome is a condition where intra-compartmental pressures increase to the point of causing a decrease in perfusion pressure, which can lead to ischemia, and if left untreated, irreversible muscle necrosis. Compartment syndrome is a clinical diagnosis. Pain out of proportion to clinical findings and pain elicited with passive extension are typical early findings, as in our patient. The classical 5 ‘P’s of compartment syndrome (pain, pulselessness, pallor, paresthesia, and paralysis) are actually signs of acute arterial obstruction, and other than pain, would be late findings 1. 

The delayed presentation of this case of compartment syndrome was atypical, as most cases occur within 24-48 hours of injury. However, bleeding disorders, such as von Willibrand disease, can contribute to delayed hematoma formation. Notably, the thigh is an unusual location for compartment syndrome. Suzuki et al reported only 8 patients with compartment syndrome of the thigh out of 3,658 blunt trauma patients seen at their institution over an 8-year period 2. Compartment syndrome involving the thigh has been associated with trauma, post-surgery (especially orthopedic or vascular surgery), tumor infiltration, exercise, snake bite, drugs, anticoagulants, and coagulopathy 3. 

Unfortunately, clinical assessment, especially based on classic signs and symptoms, has low sensitivity for the diagnosis of compartment syndrome. If the clinical assessment is equivocal, it is recommended that compartment pressures be measured. Either an absolute pressure >30 mm Hg or a delta pressure <30 mm Hg, defined as the difference between diastolic blood pressure and measured compartment pressure, should trigger emergent orthopedic consultation 1. 

Use of POCUS may facilitate the diagnosis of compartment syndrome by identifying an underlying cause. In this case, immediate detection of a large hematoma within the anterior compartment along with severe pain prompted emergent orthopedic consultation. Other case reports describe the use of ultrasound in identifying the presence of a thigh hematoma to adjunctively assist in ultimately diagnosing compartment syndrome 3, 4. These findings can also provide the clinician with confidence to expedite orthopedic consultation, especially if the orthopedic specialist is resistant or unwilling to see the patient in a timely manner. On ultrasound, hematomas typically have the appearance of rounded collections that appear hypoechoic in the acute phase and more hyperechoic with age. They should not have any Doppler flow. Ultrasound may identify alternative pathology like abscess, necrotizing fasciitis, muscle tears, myositis, or rhabdomyolysis 5. However, it is important to note that ultrasound cannot rule out compartment syndrome.

## Conclusion

Compartment syndrome can be a challenging diagnosis, especially early on in the course of illness, as many less emergent conditions can also cause extremity pain. While POCUS should not be used in isolation in the assessment of possible compartment syndrome, it can be used as an adjunct in the workup, especially if it identifies a large hematoma or other underlying cause.

## Conflicts of Interest

NL and JSA do not report any conflicts of interest. DJK is on the medical advisory board of Clarius Mobile Health.

## Supplementary Material

Video S1 Ultrasound cine clip of a large hematoma in the anterior thigh, appearing as a well circumscribed collection of mixed echogenicity.

Video S2 Ultrasound color Doppler cine clip of a large hematoma in the anterior thigh, appearing as a well circumscribed collection of mixed echogenicity without vascularity.
